# Longitudinal changes in bone mineral density among children living with HIV over 96 weeks following switch to second-line antiretroviral therapy in Uganda

**DOI:** 10.1371/journal.pgph.0005979

**Published:** 2026-02-17

**Authors:** Eva Natukunda, Erisa Mwaka, Alexander J. Szubert, Alasdair Bamford, Katja Doerholt, Centurio Wandera, Spencer Byaruhanga, Esther Nambi, Diana M. Gibb, Victor Musiime, Philippa Musoke, Ann Sarah Walker

**Affiliations:** 1 Department of Paediatrics, Joint Clinical Research Centre, Kampala, Uganda; 2 Department of Anatomy, College of Health Sciences, Makerere University, Kampala, Uganda; 3 Medical Research Council Clinical Trials Unit at University College London, London, United Kingdom; 4 Department of Paediatrics and Child Health, College of Health Sciences, Makerere University, Kampala, Uganda; 5 Makerere University-Johns Hopkins University Research Collaboration (MUJHU CARE), Kampala, Uganda; Ex DNDi, UNITED STATES OF AMERICA

## Abstract

Long-term impact of antiretroviral therapy (ART) on bone health in children living with HIV (CLWH) remains uncertain. We aimed to determine associations of change in bone mineral density (BMD) among CLWH in Uganda in a 2-year prospective sub-study in the CHAPAS-4 randomized trial (ISRCTN22964075). CLWH aged 3–15 years switched to second-line ART including tenofovir alafenamide fumarate-emtricitabine (TAF/FTC) or standard-of-care (SOC) (abacavir (ABC) or zidovudine (ZDV) with dolutegravir (DTG), atazanavir/ritonavir (ATV/r), darunavir/ritonavir (DRV/r) or lopinavir/ritonavir (LPV/r). BMD was assessed by dual-energy X-ray absorptiometry (DXA) at baseline, weeks 48 and 96 and bone turnover markers measured at baseline, week 24, 48, and 96. Robust regression analysis determined associations of BMD and bone turnover markers through week 96. Of 196 participants,167 contributed BMD measurements. Median (IQR) age was 9.9(7.0,12.3) years, 47% male, median (IQR) CD4 T-cell count 797(537,1140) cells/µl and mean (SD) viral load (log_10_ copies/ml) 4.3(0.8). Change in Procollagen type I N-terminal propeptide (PINP), C-terminal telopeptide of type I collagen (CTX) and height-adjusted (HA) BMD were similar between TAF/FTC and SOC. Greater declines in total-body-less-head (TBLH) BMD were associated with higher baseline TBLH (HA) BMD (Coef. -0.30,95% CI: -0.46, -0.15], p < 0.001) and first-line nevirapine (NVP) exposure (-0.25,95% CI: -0.43, -0.06, p = 0.009). Smaller TBLH HA BMD declines were associated with higher baseline fat-mass (0.06, 95% CI:0.01, 0.11, p = 0.021), higher lumbar spine (LS) HA BMD (0.17, 95% CI:0.03, 0.31, p = 0.015), DRV/r (0.46, p < 0.001,95% CI:0.21,0.71), DTG (0.26, p = 0.041,95% CI:0.01,0.51) or ATV/r (0.28, p = 0.026, 95% CI: 0.03, 0.52) use compared with LPV/r. Smaller declines in TBLH BMD were associated with higher baseline fat mass, higher LS HA BMD, and use of DRV/r, DTG, or ATV/r compared with LPV/r. These findings emphasize the importance of ART selection and body composition in supporting bone health among CLWH.

## Introduction

Antiretroviral therapy (ART) has transformed the lives of people living with HIV(PLWH) and significantly improved their life expectancy and quality of life. [1] However, as children with HIV (CLWH) live longer on lifelong therapy, long-term safety concerns, including effects on bone development become increasingly important. Studies have shown lower bone mineral density (BMD) among PLWH compared with their negative counterparts [2]. The prevalence of low BMD among adults living with HIV is 20–80% [3,4], while in CLWH ranges from 4-32% with the lower prevalence in high-income countries [5] and higher in low and middle-income countries in sub-Saharan Africa and Asia, where stunting, undernutrition, and delayed puberty are common [6,7]. Achieving optimal peak bone mass during childhood and adolescence is crucial for reducing the risk of osteoporosis and fractures later in life. Yet, HIV infection and ART exposure may disrupt normal bone accrual during the developmental window [6].

Low BMD in HIV is influenced by a combination of traditional risk factors such as low body mass index (BMI), female sex, reduced physical activity, and HIV related mechanisms like immune activation and direct drug-induced toxicity. [8,9] In addition, body composition, such as fat mass and lean mass, have an impact on bone development among children, given the relationship between adiposity, lean tissue, and BMD [10]. Despite the existing literature on risk factors for low BMD in PLWH, the associations of change in BMD in CLWH switching to second-line ART remain underexplored in resource-limited settings (RLS).

Long-term use of older generation boosted protease inhibitors (bPIs), such as ritonavir-boosted lopinavir(LPV/r), have been associated with deterioration in bone health [11]. Previous studies have demonstrated that tenofovir disoproxil fumarate (TDF) may contribute to reduced BMD and alterations in bone turnover markers (BTMs) [6,12]. These findings have raised concerns about the long-term effects of TDF on bone health and signify the importance of identifying alternative treatment strategies that minimize bone loss and optimize peak bone mass in childhood.

Circulating BTMs, procollagen type 1 N-terminal propeptide (P1NP), and C-terminal telopeptide of type 1 collagen (CTX) released during bone remodeling can be a useful tool in predicting fracture risk among adults with low BMD but there is limited literature on their usefulness in predicting BMD change among CLWH [13]. Among South African CLWH with viral suppression, annual percentage change in P1NP was positively associated with BMD [14]. An adult study found elevated BTMs among PLWH with viral suppression who switched to TDF compared with ABC [15].

Tenofovir alafenamide fumarate (TAF), a novel prodrug of tenofovir, exhibits high intracellular conversion to its active form, resulting in potent antiviral efficacy at lower doses compared to TDF [16]. Although TAF has demonstrated improved renal and bone safety compared with TDF in adults and adolescents [17,18], its long-term effects in younger children remain unclear. Few studies have evaluated longitudinal changes in BMD and BTMs following ART switch in African pediatric populations, where limited access to dual-energy X-ray absorptiometry (DXA) and a lack of reference data complicates the assessment. Moreover, the influence of previous first-line non-nucleoside reverse transcriptase inhibitors NNRTIs) exposure as well as second-line anchor drugs on bone outcomes remain underexplored.

Although several studies have investigated the impact of TAF on bone health among the adult populations [19,20], there is a paucity of data regarding its effects on BMD and BTMs in CLWH switching to second-line ART, when co-administered with bPIs or dolutegravir (DTG) [17,18,21]. This sub-study aimed to determine the associations of BMD and BTM changes among CLWH switching to second-line TAF/FTC or standard- of- care (SOC) based backbone when co-administered with DTG or bPIs. We aimed to identify modifiable risk factors for bone loss in CLWH from low-income countries where diagnostic facilities are limited.

## Materials and methods

### Ethics statement

The main CHAPAS-4 trial was approved by the JCRC institutional review board (Reference -JC 1417), Uganda National Council of Science and Technology (UNCST)(HS:2369), and National Drug Authority (Ref CTC 066). Written informed consent was obtained from the caregivers or parents of all participants, and written assent was obtained from the children aged 8 years and above before enrolment into the sub-study.

### Study design

**Fig 1:**
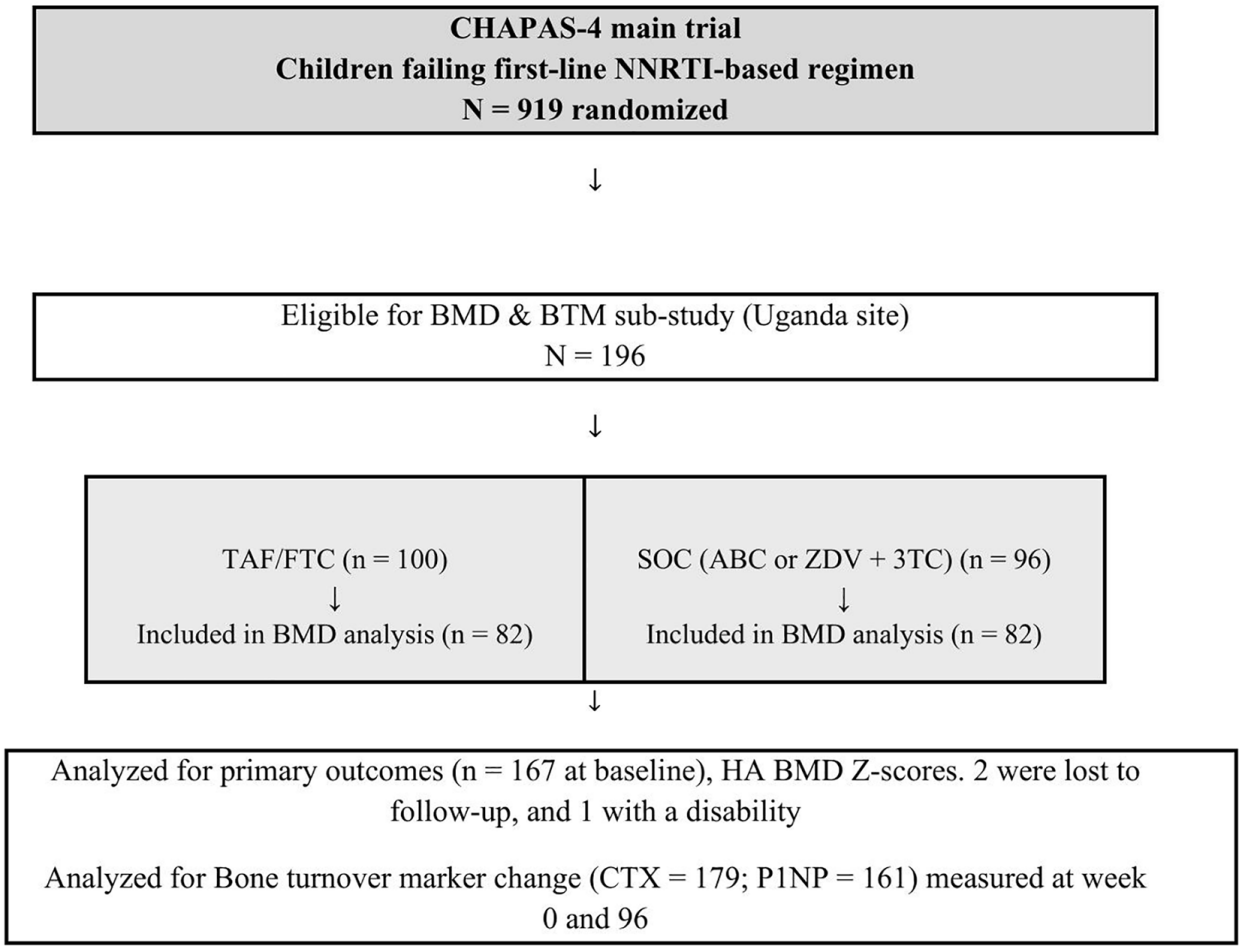
Consort flow diagram.

**Fig 2:**
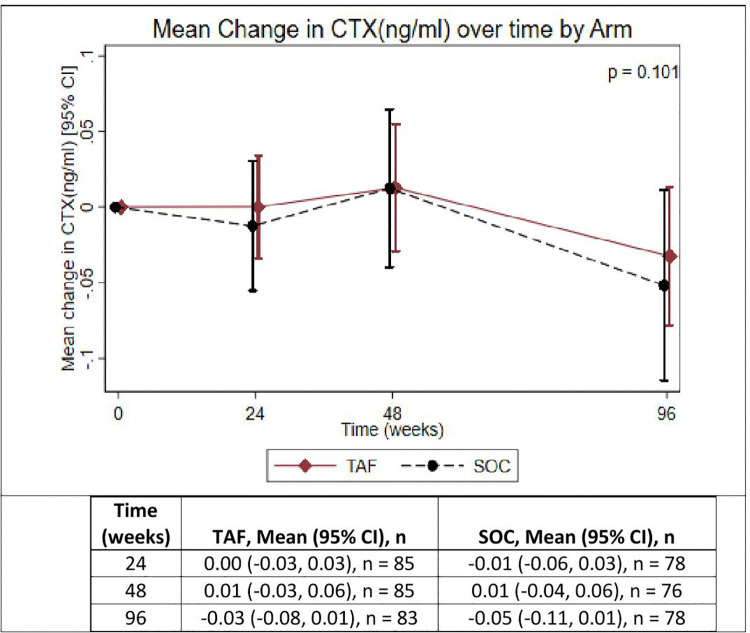
Mean change in C-terminal telopeptide (ng/ml) over time in participants receiving TAF/FTC or SOC-based backbone. ng/ml = nanograms per ml, TAF/FTC = Tenofovir alafenamide fumarate/Emtricitabine, SOC = Standard of care, CTX = C-Terminal Telopeptide of type I collagen.

**Fig 3:**
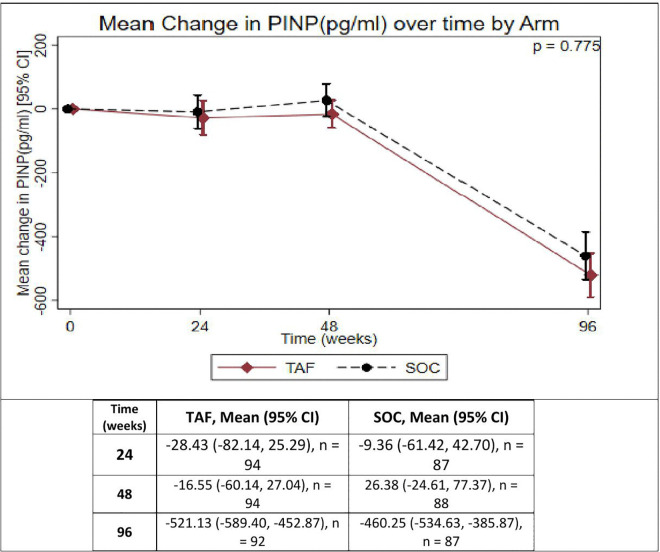
Mean change in procollagen type I N-terminal propeptide (pg/ml) over time in participants receiving TAF/FTC or SOC-based backbone. pg/ml = picograms per ml, TAF/FTC = Tenofovir alafenamide fumarate/Emtricitabine, SOC = Standard of care P1NP=Procollagen Type I N-terminal Propeptide.

**Fig 4:**
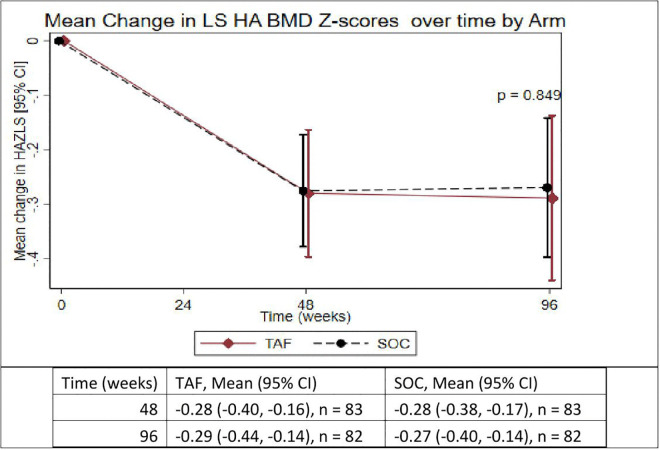
Mean change in height-adjusted lumbar spine BMD Z-scores (LS HA BMD) over time in participants on TAF/FTC or SOC. TAF = Tenofovir alafenamide fumarate, SOC = Standard of care.

**Fig 5:**
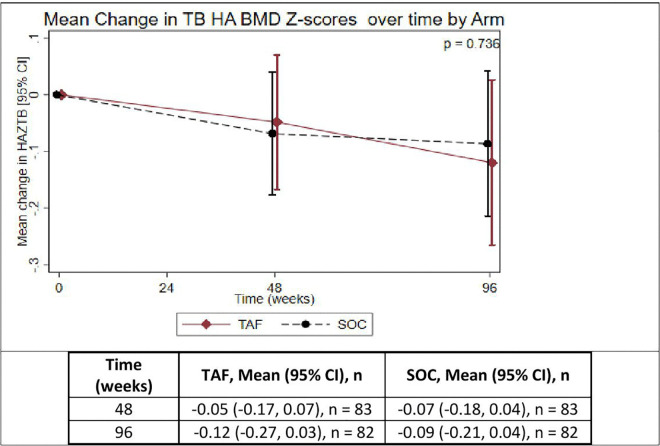
Mean change in height-adjusted total body less head BMD Z-scores (TBLH HA BMD) over time in participants on TAF/FTC or SOC. TAF = Tenofovir alafenamide fumarate, SOC = Standard of care.

We conducted a prospective longitudinal sub-study from January 2019 to March 2023 at the Joint Clinical Research Centre (JCRC), Uganda. We enrolled the first participant on 21^st^ Jan 2019 and the last on 23^rd^ Feb 2021.One hundred and ninety-six children aged 3–15 years were enrolled in this sub-study, which was nested within the main CHAPAS-4 trial, a randomized controlled trial (ISRCTN22964075) that enrolled children who were failing non-nucleoside reverse transcriptase inhibitor (NNRTI) based first-line ART in Uganda, Zambia and Zimbabwe. Participants were randomized to receive TAF/FTC or standard-of-care (SOC)-based backbone, which was ABC/3TC (lamivudine) or ZDV/3TC (whichever was not used first-line), and simultaneously randomized to DTG, ATV/r, LPV/r, or DRV/r as anchor drug. All children were followed up for 96 weeks. In the sub-study, we assessed associations of longitudinal change in BMD and BTMs among CLWH on TAF/FTC or SOC backbone (Fig 1–5).

### Study population

Children aged 3–15 years, switching to second-line ART were eligible for inclusion in the sub-study. Participants were recruited from the JCRC pediatric outpatient clinic and nearby public health facilities. We excluded one participant with disabilities that compromised body positioning during scan acquisition.

### Measurements

Areal BMD was assessed using DXA Hologic Discovery Wi apex software version 3.1 (Hologic Bedford Inc., Bedford, MA, USA) at baseline, weeks 48 and 96 at one facility with the same densitometer. The procedure for BMD acquisition has been previously described [22]. Areal BMD values underestimate true volumetric BMD in smaller bones [23,24]. We therefore determined height-adjusted BMD (HA BMD) Z-scores using reference data as per International Society of Clinical Densitometry (ISCD) recommendation [25,26]. Plasma samples were collected at baseline, weeks 24, 48, and 96, and stored at -80°C. Samples were obtained from fasting participants during morning hours (mean collection time ≈ 10:30 a.m., SD 1 hour) to minimize the effect of diurnal variation for C-terminal telopeptide of type I collagen (CTX). CTX (0.156-10ng/ml) was measured in nanograms per litre (ng/ml)and Procollagen type I N-terminal propeptide (P1NP)(78–5000pg/ml) in picograms per millilitre (pg/ml) using enzyme-linked immunosorbent assay (ELISA) following the manufacturer’s manual [27]. Each sample was run in duplicate and an average reading was obtained.

Collection of baseline demographic and clinical characteristics, including age, sex, time since HIV diagnosis, previous ART history, WHO stage, weight and height, have been previously described along with baseline viral load, CD4 count [22]. Data on BMD and BTMs were collected and recorded by trained personnel blinded to the treatment arms.

### Statistical analysis

All analyses were performed using Stata 15 (Stata Corp LLC, College Station, Texas). Baseline characteristics were summarized as means and standard deviations (SD) for continuous, normally distributed data (assessed using the Shapiro-Wilk test) and as medians (interquartile ranges) for non-normally distributed data. Categorical data were presented as frequencies and percentages.

The primary outcomes were changes in HA BMD Z-scores—total body less head (TBLH) and lumbar spine (LS), and bone turnover markers (P1NP and CTX) from baseline (Week 0) to Week 96. Changes were calculated as the difference between values at Week 96 and baseline. Participants with missing baseline values were excluded from analysis as their change could not be calculated. Z-scores were approximately normally distributed so were modelled on the absolute scale whereas BTMs were approximately log-normally distributed so were modelled on the log scale.

Group comparisons were conducted to assess differences in changes in HA BMD Z-scores and BTMs between the TAF/FTC and SOC treatment groups. Independent t-tests were used for normally distributed changes, while Wilcoxon rank-sum tests were applied for non-normally distributed changes.

To identify baseline factors associated with the four outcomes above, robust regression was employed to reduce the influence of outliers and potential heteroscedasticity, providing more reliable estimates than a normal linear regression model under these conditions. Models assessed associations of 96-week changes in HA BMD Z-scores and BTMs, adjusting for baseline log_10_ viral load, CD4 count, BMI Z-score, fat mass, fat-free mass, P1NP, CTX, first-line ART regimen, WHO stage, sex, treatment arm, and randomized anchor and backbone drugs. Log transformations were applied to viral load, CD4 count, P1NP, and CTX to reduce right-skewness and stabilize variance, and age at the measurement was excluded as it is numerically equal to age at ART initiation plus time on first-line ART; a priori because the Z-score calculation includes current age and current age is not modifiable we chose to estimate the effects of age at ART initiation and time on first-line ART. Model adequacy was evaluated by regressing the residuals from each robust regression model within a standard linear regression framework to assess whether any systematic variation remained.

The sub-study was nested within the CHAPAS-4 trial; therefore, the sample size was determined by the parent trial enrolment, and no formal a priori sample size calculation was conducted. Based on the observed sample sizes and standard deviations, the study had 80% power to detect the differences in the outcomes between TAF/FTC and SOC arms at 5% significance level.

To visualize trends, line graphs with 95% confidence intervals were generated for mean changes in HA BMD Z-scores, P1NP, and CTX over 96 weeks, stratified by treatment group (TAF/FTC vs. SOC). A box plot illustrated the impact of prior nevirapine (NVP)-based vs. efavirenz (EFV)-based first-line regimens on baseline HA BMD and also on changes at week 96.

## Results

The baseline characteristics are shown in **Table 1**. One hundred ninety-six children aged 3–15 years were enrolled in the sub-study, of which 167 had BMD measurements. There was no significant difference between the baseline characteristics of participants receiving SOC or TAF-based backbone.

**Table 1:** Baseline characteristics study participants N = 196.

	SOC arm = 96	TAF/FTC arm = 100	Total = 196
**Characteristic**			
Age: median (IQR)	9.9 (7.1,12.3)	9.9 (6.9,12.2)	9.9 (7.0,12.3)
Male sex (n%)	45 (46)	48 (48)	93 (47)
**Anthropometry**			
Weight for age Z score (WAZ) mean (SD)	−1.18 (1.29)	−1.56 (1.22)	−1.37 (1.27)
Height-for-age-Z-score (HAZ) median (IQR)	−1.12 (−1.59,0.13)	−1.05 (−1.62, −0.20)	−1.09 (−1.60, −0.06)
BMI-for-age-Z-score (BAZ) median (IQR)	−1.15 (−1.74, −0.45)	−1.23 (−2.12, −0.57)	−1.20 (−1.99, −0.51)
Fat-free mass (kg)(IQR)	22.83 (18.00,29.25)	22.50 (17.60,27.20)	22.80 (17.80,28.00)
Muscle mass(kg)(IQR)	21.58 (17.03, 27,7)	21.30 (16.70,25.80)	21.45 (16.70,26.60)
Fat mass(kg)median (IQR)	3.28 (2.58,4.25)	2.90 (2.10,3.90)	3.10 (2.40,4.10)
**HIV characteristics**			
CD4 cell count(cells/ul): median (IQR)	780.5 (514.5,1065)	808.5 (548,1172.5)	797 (537,1140)
CD4%, median (IQR)	31 (23,36.5)	32 (23.5,37)	31 (23,37)
VL (log10copies/ml) mean (SD)	4.25 (0.75)	4.26 (0.80)	4.26 (0.77)
**WHO Stage (%)**			
Stage 1&2	87 (90.6)	91 (91)	178 (90.8)
Stage 3&4	9 (9)	9 (9)	18 (9)
**First line ART regimen N (%)**			
EFV-based	42	54	96 (48)
NVP-based	58	46	104 (52)
Age at First-line ART initiation(years): median (IQR)	4.18 (1.67,6.45)	4.19 (2.07,7.35)	4.18 (1.88,7.02)
First-line ART duration in years: median (IQR)	5.2 (3.6,6.8)	4.8 (2.9,6.70)	5.0 (3.0,6.7)
**HA BMD Z-scores(N = 167)**			
LS: mean (SD)	−1.02 (1.00)	−1.20 (0.87)	−1.11 (0.94)
TBLH: median (IQR)	−1.17 (−1.72, −0.49)	−1.57 (−1.95, −1.02)	−1.39 (−1.90, −0.81)
**Bone turnover markers (N = 183)**			
CTX (ng/ml), median (IQR)	0.24 (0.13,0.43)	0.24 (0.16,0.40)	0.24 (0.16,0.40)
P1NP (pg/ml), median (IQR)	704 (553,944)	858 (574,1031)	785 (560,1014)

Note: missing data due to scans not being able to be scheduled during the baseline window due to machine availability, assay failure and failure to store plasma.

Abbreviations: ART = antiretroviral therapy, IQR = interquartile range, BMI = body mass index, EFV = efavirenz, NVP = Nevirapine, LS = lumbar spine, TBLH = total body less head, HA BMD Z scores = Height adjusted bone mineral density Z- score, CTX = C-terminal telopeptide of type 1 collagen, P1NP = Procollagen type I N-terminal propeptide, VL = viral load, TAF/FTC = tenofovir alafenamide fumarate/emtricitabine, SOC = standard of care.

**Table 2** summarizes the change in HA BMD Z-scores. Changes in HA BMD Z-scores over 96 weeks were comparable between TAF/FTC and SOC. The mean changes in TBLH HA BMD Z-scores were slightly greater in the TAF/FTC group compared to SOC; however, the difference was not statistically significant (p = 0.736). Similarly, LS HA BMD Z-scores declined in both groups with no significant difference observed (p = 0.849).

**Table 2:** Height adjusted BMD Z scores and bone turnover marker change at 96 weeks by treatment group (TAF vs. SOC).

Variable	Sample size(N)		mean (SD)		P-value
	SOC	TAF/FTC	SOC	TAF/FTC	
**Changes in HA BMD Z-scores from baseline to week 96**					
TBLH	82	82	−0.09 (0.59)	−0.12 (0.67)	0.736
LS	82	82	−0.27 (0.59)	−0.29 (0.70)	0.849
**Changes in BTMs from baseline to week 96**					
			**Median (IQR)**		
P1NP (pg/ml)	78	83	−379.3 (−627.2, −209.6)	−496 (−718.05, −269.7)	0.775
CTX (ng/ml)	87	92	−0.02 (−0.11, 0.06)	−0.03 (−0.11,0.04)	0.101

LS **=** Lumbar spine, TBLH **=** Total body less head, HA BMD = Height adjusted bone mineral density, SOC = Standard of care, TAF/FTC = Tenofovir alafenamide fumarate/emtricitabine, CTX = C-terminal Telopeptide of type 1 collagen, P1NP = Procollagen Type 1 N-terminal Propeptide.

Bone turnover markers (BTMs) also showed similar patterns of decline between groups. The median change in P1NP levels was slightly larger in the TAF/FTC group compared to SOC, but this difference was not significant (p = 0.775). Likewise, CTX levels decreased in both groups with no significant difference between TAF/FTC and SOC (p = 0.101).

Multivariate analysis results for BMD associations are presented in S1 Table. Every one-unit kg higher baseline fat mass was associated with a 0.06 SD higher in TBLH HA BMD Z-scores at week 96. Similarly, every unit higher baseline LS HA BMD Z-score was associated with a 0.17 unit increase in baseline HA BMD Z-score. Every unit higher TBLH HA BMD Z-score was associated with a 0.30-unit greater decline in TBLH HA BMD Z-score at week 96. First-line NVP use was associated with a 0.25-unit greater decrease in TBLH HA BMD Z-score decline compared with first-line EFV use. A similar trend was observed for LS HA BMD Z-scores, with a 0.30-unit greater decrease in LS HA BMD Z-score associated with first-line NVP use. ATV/r based second line regimen was associated with a 0.25 SD smaller TBLH HA BMD Z-score decline compared with LPV/r, as were DRV/r and DTG.

Multivariate analysis results for BTM associations are presented in S2 Table. A 1% higher baseline CTX was associated with a 0.32% decline in CTX level, while a 1% higher baseline P1NP was associated with a 0.31% decline in CTX level. Similarly, a 1% higher baseline P1NP was associated with a 1.07% decline in P1NP level. Prior exposure to first-line NVP was associated with a 25% higher CTX level compared to prior EFV-based regimens. Female sex was associated with 20% higher CTX levels compared to males.

## Discussion

In this two-year sub-study of the CHAPAS-4 clinical trial among children randomized to TAF/FTC or SOC-based backbone after first-line NNRTI-based ART failure, the independent associations with BMD decline included prior first-line NVP exposure and higher baseline TBLH HA BMD. In addition, receiving DRV/r, ATV/r or DTG compared with LPV/r, higher baseline fat-mass, and higher baseline LS BMD were associated with a greater increase in BMD. BTM parameters showed no significant difference between TAF/FTC and SOC arms.

Previous adult and adolescent studies have reported a mixture of traditional and HIV associated risk factors for BMD decline [28]. In our study, prior use of NVP-based first-line ART was associated with greater BMD decline compared with EFV. One possible explanation for this observation, although hypothetical, is that NVP may have relatively bone-sparing effects compared to EFV and some second-line ART regimens. Previous studies have reported that NVP may have neutral or suppressive effects on cytokine interleukin (IL)-6 release, which promotes osteoclast activity, whereas EFV has been associated with increased cytokine release, which may enhance bone turnover [29,30]. Although NVP is no longer commonly used, children transitioning to currently recommended regimens from NVP may be at risk of reduced BMD accrual since the putative bone-sparing effects of NVP were not sustained when CLWH transitioned to second-line ART regimens. These explanations remain theoretical, and interventional studies are required to confirm these proposed pathways.

The anchor drugs (ATV/r, DRV/r, and DTG but not LPV/r) were significantly associated with an increase in TBLH HA BMD. Compared with LPV/r, mean increases in TBLH Z-scores were 0.28 SD higher with ATV/r, 0.46 SD higher with DRV/r, and 0.26 SD higher with DTG**.** This finding is consistent with results from previous adult studies. In the CASTLE sub-study, hip BMD declined by –2.4% with LPV/r versus –1.2% with ATV/r after 48 weeks [31], Similarly, Brown et al. reported comparable bone outcomes for ATV/r and DRV/r in adults initiating TDF-based ART, with mean total hip BMD declines of –2.7% and –2.4%, respectively, at week 48 [32]. The absence of a significant increase in TBLH BMD with LPV/r may be attributed to the greater bone loss associated with ritonavir (RTV) boosting [33], which is more pronounced with LPV/r than with ATV/r or DRV/r. In addition, Bonfanti et al. demonstrated significant bone recovery after switching from PI-based to DTG-based therapy, with LS BMD increasing by 1.7% and femoral neck by 1.3% over 48 weeks, accompanied by reductions in BTMs, hence demonstrating a positive impact on BMD in real-world adult cohorts, further supporting its bone-sparing properties [34].

In our cohort, fat mass was positively associated with total body less head (TBLH) BMD, with a mean increase of 0.06 SD per unit increase in fat mass, whereas the corresponding association with lumbar spine (LS) BMD was smaller and non-significant (0.04 SD per unit). This is similar to a previous report of fat mass correlation with bone site that was stronger with TBLH BMD than LS BMD (p = 0.001) [35]. Fat mass contributes to the overall body weight, which increases mechanical loading on weight-bearing bones. The relationship between fat mass and bone density may be stronger at weight-bearing sites compared to the spine, which has more trabecular bone and is less influenced by mechanical loading [36].

Higher baseline TBLH HA BMD Z-scores was associated with -0.3SD decline in TBLH HA BMD Z-scores over 96 weeks. Comparative literature is scarce; however, in a previous study among virally suppressed South African CLWH, there was a progressive annual decline of -0.05 SD in TBLH HA BMD Z-scores over time despite viral suppression [14]. The greater decline in our cohort likely reflects the negative impact of viral non-suppression and inflammation on bone formation, compounded by delayed growth recovery after ART switch.

LS predominantly consists of trabecular bone, which is more metabolically active and responds more rapidly to changes in interventions compared with TBLH, which is cortical [37]. The observed LS HA BMD Z-scores prediction may reflect improvement in BMD due to reduced HIV-related bone resorption or direct effects of effective second-line ART.

In our study, we observed that there was a decline in BTMs by week 96, suggesting a reduction in osteoblast and osteoclast activity. The decline could be attributed to multiple factors. This trend may be attributed to growth. Bone remodeling declines with age and increases up to mid-puberty, followed by another decline [38,39]. The median age of our study participants was approximately 10 years at baseline, meaning they were approaching their peak growth period, particularly the females. By week 96, growth velocity may have slowed, leading to reduced BTMs; we did not do a Tanner evaluation to confirm the pubertal status either at baseline or over follow-up. One limitation is therefore that we are not able to formally assess the impact of puberty on these changes; however, power would be very low to detect differences in the effect of backbone NRTIs across pubertal stages. Some children may have reached late puberty, where BTMs naturally declined.

The decline in BTMs may also be attributed to a reduction in systemic inflammation following viral suppression, although a reduction in bone turnover may also occur when there is viral suppression [40]. In the CHANGES longitudinal study among CLWH, there was a mean increase in BTMs over time, contrary to our findings. This discrepancy may be due to the study enrolling predominantly younger children with viral suppression [14]. Longer periods of viral suppression may lead to an increase in BTMs.

In our study, PINP changes between TAF and SOC were comparable (–496 pg/mL vs –379 pg/mL). Although direct comparative paediatric data on BTMs between TAF and ABC are limited, Winston et al. reported no significant differences in hip and spine BMD at week 48 (0.27% vs 0.16% at the hip, and 0.10% vs 0.03% at the spine) between adults maintained on ABC/3TC and those switched to TAF/FTC [41]. In addition, a study comparing bone outcomes in ART-naïve adults who started TAF, TDF, or ABC-based first-line ART found no significant difference in BTMs between participants on TAF or ABC by week 48 **(**18 pg/mL for TAF, and 21 pg/mL for ABC), which is consistent with our findings [42].

Higher BTMs at baseline were associated with a greater decline in BTMs after the switch to effective second-line ART. This may be explained by a possible significant reduction in systemic inflammation compared with those who had a lower level of baseline BTMs.

There are several limitations to this study. A relatively short follow-up period and hence long-term effects of ART on bone outcomes may not have been captured, given the fact that TBLH is primarily trabecular, hence less sensitive to changes following drug exposure. Secondly, the study did not collect data on pubertal development, which is a crucial contributor to bone health. The potential for confounding due to the low vitamin D status that is prevalent in Ugandan children: Vitamin D was not measured, and neither was dietary calcium intake nor sun exposure, which are all determinants for bone mineralization [43–45]. We did not collect data on physical activity, one of the strongest predictors of peak bone mass development, and therefore its impact was not assessed [46,47]. The study was conducted at a single site, which limits the generalizability of the findings.

The sample sizes and observed variability indicated that the study had sufficient power to detect moderate between-group differences in BMD and BTM-related outcomes, but limited power to detect smaller differences in the BMD and BTM-outcomes. In addition, we used HA BMD reference values from the black American population, which may be inappropriate given the lack of local reference values [25].

Despite the limitations, this is the first study to determine associations of bone changes among CLWH switching to second-line ART in Uganda. The longitudinal design allowed for detailed tracking of changes in BTMs over time, and the inclusion of multiple ART regimens provided a detailed understanding of the comparative bone changes.

In conclusion**,** we observed no difference in the BTMs or HA BMD between children and adolescents receiving TAF/FTC and SOC-based backbone. Fat mass, second-line DRV/r, ATV/r, or DTG use (but not LPV/r), and higher baseline LS BMD were associated with an increase in BMD among CLWH. Prior first-line NVP exposure was associated with BMD decline. Optimizing drug choices to minimize effects on body composition alongside nutritional programs is crucial for promoting bone health in this population.

## Supporting information

S1 Fig Box plot of lumbar spine (LS) and total-body-less-head (TBLH) height-adjusted BMD Z-scores stratified by first-line ART regimen (EFV vs. NVP) at baseline. The baseline LS HA BMD Z scores for prior first-line EFV exposure were significantly lower than those on NVP, with a mean difference of -0.33 (95% CI: -0.64 to -0.03, p = 0.034). In contrast, no significant difference was observed in the TBLH BMD Z scores between the two groups (z = -1.86, p = 0.063). Participants who were previously on NVP showed a greater decline, as the majority experienced negative changes in LS HA BMD Z-scores compared to those on EFV-based regimens. EFV = Efavirenz, NVP = Nevirapine.
(TIF)

S2 Fig The boxplot illustrates the change in LS HA BMD and TBLH HA BMD Z-score changes over 96 weeks among participants previously on EFVor NVP-based first-line ART regimens. The mean HA LS HA BMD Z score change was -0.118 (SD = 0.567) in the EFV-based and -0.437 (SD = 0.675) in the NVP-based, with a statistically significant mean difference of 0.320 (95% CI: 0.110, 0.530), p = 0.003. For HA TB BMD Z-score change, the mean was -0.006 (SD = 0.583) in the EFV group and -0.199 (SD = 0.673) in the NVP group, with a non-significant mean difference of 0.193 (95% CI: -0.019, 0.405), p = 0.074. EFV = Efavirenz, NVP = Nevirapine.
(TIF)

S1 Table Multivariable linear regression analysis identifying factors associated with a change in bone mineral density. (DOCX)

S2 Table Multivariable linear regression analysis identifying factors associated with change in bone turnover markers. (DOCX)
