# Supplementary material for: Longitudinal changes in bone mineral density among children living with HIV over 96 weeks following switch to second-line antiretroviral therapy in Uganda
Source: PLOS Glob Public Health. 2026 Feb 17;6(2):e0005979. doi: 10.1371/journal.pgph.0005979 (PMC12912597; doi:10.1371/journal.pgph.0005979)
Supplement: S1 Table — (DOCX) [file pgph.0005979.s003.docx]

**S1 Table. Multivariable linear regression analysis identifying factors associated with a change in bone mineral density.**

|  | **Change in TBLH height adjusted BMD Z-scores from baseline to week 96, n=156** | | **Change in LS height adjusted BMD Z-score from baseline to week 96, n=156** | |
| --- | --- | --- | --- | --- |
|  | **Coef. [95% CI]** | **P-value** | **Coef. [95% CI]** | **P-value** |
| **Baseline factors** |  |  |  |  |
| VL (log10copies/ml) | 0.03[-0.03,0.08] | 0.308 | -0.02[-0.08,0.04] | 0.518 |
| CD4 cell count(cells/ml) | 0.03[-0.08,0.13] | 0.584 | 0.08[-0.03,0.19] | 0.165 |
| Time on first-line ART (years) | -0.02[-0.11,0.07] | 0.646 | 0.01[-0.09,0.11] | 0.879 |
| Age at first-line ART initiation(years) | -0.005[-0.08,0.07] | 0.903 | 0.00[-0.09,0.08] | 0.964 |
| Fat-mass (kg) | **0.06[0.01,0.11]** | **0.021** | 0.04[-0.02,0.09] | 0.189 |
| BMIZ | -0.04[-0.17,0.08] | 0.504 | 0.02[-0.12,0.16] | 0.793 |
| Fat-free-mass (kg) | 0.003[-0.03,0.04] | 0.862 | -0.003[-0.04,0.03] | 0.87 |
| **LS HA BMD** | **0.17[0.03,0.31]** | **0.015** | -0.13[-0.28,0.02] | 0.096 |
| **TBLH HA BMD** | **-0.30[-0.46, -0.15]** | **<0.001** | -0.07[-0.24,0.10] | 0.433 |
| **First line ART regimen** |  |  |  |  |
| EFV based | 1 | 1 | 1 | 1 |
| NVP based | **-0.25[-0.43, -0.06]** | **0.009** | **-0.30[-0.50, -0.10]** | **0.004** |
| **WHO stage** |  |  |  |  |
| Stage 1& 2 | 1 | 1 | 1 | 1 |
| Stage 3&4 | 0.04[-0.25,0.34] | 0.779 | 0.05[-0.27,0.37] | 0.761 |
| **Sex** |  |  |  |  |
| Male | 1 | 1 | 1 | 1 |
| Female | 0.00[-0.18,0.19] | 0.963 | 0.06[-0.14,0.27] | 0.544 |
| **Backbone(randomized)** |  |  |  |  |
| SOC | 1 | 1 | 1 | 1 |
| TAF/FTC | -0.12[-0.29,0.05] | 0.177 | -0.06[-0.25,0.13] | 0.552 |
| **Anchor drug(randomized)** |  |  |  |  |
| LPV/r | 1 | 1 | 1 | 1 |
| ATV/r | **0.28[0.03,0.52]** | **0.026** | 0.003[-0.26,0.27] | 0.981 |
| DRV/r | **0.46[0.21,0.71]** | **<0.001** | 0.06[-0.21,0.34] | 0.646 |
| DTG | **0.26[0.01,0.51]** | **0.041** | -0.06[-0.33,0.22] | 0.677 |

ART=Antiretroviral therapy**,** BMIZ=Body mass index, TAF/FTC=Tenofovir alafenamide fumarate/emtricitabine, SOC=Standard of care, EFV=Efavirenz, NVP=Nevirapine, DTG=Dolutegravir, ATV/r=Atazanavir/ritonavir, DRV/r=Darunavir/ ritonavir, Lopinavir/ritonavir, TBLH HA BMD=Height adjusted total body less head BMD Z-score, LS HA BMD =Height adjusted lumbar spine BMD Z-score. No evidence of non-linearity of effects of continuous variables (tested using multivariable factorial polynomials (Stata mfp)
