# Supplementary material for: Longitudinal changes in bone mineral density among children living with HIV over 96 weeks following switch to second-line antiretroviral therapy in Uganda
Source: PLOS Glob Public Health. 2026 Feb 17;6(2):e0005979. doi: 10.1371/journal.pgph.0005979 (PMC12912597; doi:10.1371/journal.pgph.0005979)
Supplement: S2 Table — (DOCX) [file pgph.0005979.s004.docx]

**S2 Table. Multivariable linear regression analysis identifying factors associated with change in bone turnover markers**

|  | **Change in log _10_ CTX from baseline to week 96, n= 151** | | **Change in log _10_ P1NP from baseline to week 96**, **n= 152** | |
| --- | --- | --- | --- | --- |
|  | **Coef. [95% CI]** | **P-value** | **Coef. [95% CI]** | **P-value** |
| **Baseline predictors** |  |  |  |  |
| VL (log10copies) | 0.00[-0.05,0.05] | 0.976 | 0.01[-0.02,0.05] | 0.459 |
| CD4 cell count(cells/ul) | -0.05[-0.15,0.05] | 0.310 | -0.02[-0.09,0.04] | 0.49 |
| Time on first-line ART (years) | 0.03[-0.06,0.11] | 0.514 | 0.01[-0.04,0.07] | 0.642 |
| Age at first-line ART initiation(years) | 0.07[-0.01,0.14] | 0.079 | 0.01[-0.04,0.06] | 0.737 |
| Fat-mass(kg) | -0.05[-0.10,0.00] | 0.062 | -0.01[-0.04,0.03] | 0.721 |
| BMIZ | 0.01[-0.11,0.14] | 0.833 | -0.04[-0.13,0.04] | 0.338 |
| Fat-free-mass(kg) | 0.02[-0.01,0.05] | 0.313 | 0.01[-0.01,0.03] | 0.555 |
| CTX (ng/ml) | **-0.32[-0.44, -0.21]** | **<0.001** | 0.03[-0.17,0.24] | 0.742 |
| P1NP (pg/ml) | **-0.31[-0.54, -0.09]** | **0.007** | **-1.07[-1.22, -0.93]** | **<0.001** |
| **First line ART regimen** |  |  |  |  |
| EFV based | 1 | 1 | 1 | 1 |
| NVP based | **0.25[0.08,0.43]** | **0.005** | 0.01[-0.12,0.11] | 0.918 |
| **WHO stage** |  |  |  |  |
| Stage 1& 2 | 1 | 1 | 1 | 1 |
| Stage 3&4 | -0.03[-0.32,0.26] | 0.824 | -0.05[-0.24,0.13] | 0.556 |
| **Sex** |  |  |  |  |
| Male | 1 | 1 | 1 | 1 |
| Female | 0.20[-0.02,0.39] | 0.029 | 0.02[-0.09,0.14] | 0.7 |
| **Backbone** |  |  |  |  |
| SOC | 1 | 1 | 1 | 1 |
| TAF/FTC | 0.02[-0.15,0.18] | 0.837 | 0.00[-0.11,0.10] | 0.937 |
| **Anchor drug** |  |  |  |  |
| LPV/r | 1 | 1 | 1 | 1 |
| ATV/r | 0.01[-0.22,0.25] | 0.915 | 0.00[-0.15,0.15] | 0.981 |
| DRV/r | -0.04[-0.27,0.19] | 0.723 | 0.10[-0.04,0.24] | 0.163 |
| DTG | 0.01[-0.22,0.25] | 0.918 | -0.03[-0.18,0.11] | 0.650 |

ART=Antiretroviral therapy, BMIZ=Body mass index, TAF/FTC=Tenofovir alafenamide fumarate/emtricitabine, SOC=Standard of care, EFV=Efavirenz, NVP=Nevirapine, DTG=Dolutegravir, ATV/r=Atazanavir/ritonavir, DRV/r=Darunavir ritonavir, Lopinavir/ritonavir, CTX=C-Telopeptide, P1NP= Procollagen **Type I N-terminal Propeptide. No evidence of non-linearity of effects of continuous variables (tested using multivariable fractional polynomials (Stata mfp)**
